# Selection and Characterization of YKL-40-Targeting Monoclonal Antibodies from Human Synthetic Fab Phage Display Libraries

**DOI:** 10.3390/ijms21176354

**Published:** 2020-09-01

**Authors:** Kyungjae Kang, Kicheon Kim, Se-Ra Lee, Yoonji Kim, Joo Eon Lee, Yong Sun Lee, Ju-Hyeon Lim, Chung-Su Lim, Yu Jung Kim, Seung Il Baek, Du Hyun Song, Jin Tae Hong, Dae Young Kim

**Affiliations:** 1 New Drug Development Center, Osong Medical Innovation Foundation, Cheongju-si, Chungcheongbuk-do 28160, Korea; kyungjae@kbiohealth.kr (K.K.); srlee@kbiohealth.kr (S.-R.L.); yoon555@kbiohealth.kr (Y.K.); jooeona@kbiohealth.kr (J.E.L.); sinistemcells@kbiohealth.kr (J.-H.L.); opern88@kbiohealth.kr (C.-S.L.); yjkim@kbiohealth.kr (Y.J.K.); bsi022013@kbiohealth.kr (S.I.B.); biosong@kbiohealth.kr (D.H.S.); 2 College of Pharmacy, Chungbuk National University, Cheongju-si, Chungcheongbuk-do 28160, Korea; k.kicheon@gmail.com (K.K.); kallintz@gmail.com (Y.S.L.)

**Keywords:** YKL-40, CHI3L1, monoclonal antibody, phage display, lung metastasis

## Abstract

YKL-40, also known as chitinase-3-like 1 (CHI3L1), is a glycoprotein that is expressed and secreted by various cell types, including cancers and macrophages. Due to its implications for and upregulation in a variety of diseases, including inflammatory conditions, fibrotic disorders, and tumor growth, YKL-40 has been considered as a significant therapeutic biomarker. Here, we used a phage display to develop novel monoclonal antibodies (mAbs) targeting human YKL-40 (hYKL-40). Human synthetic antibody phage display libraries were panned against a recombinant hYKL-40 protein, yielding seven unique Fabs (Antigen-binding fragment), of which two Fabs (H1 and H2) were non-aggregating and thermally stable (75.5 °C and 76.5 °C, respectively) and had high apparent affinities (*K*_D_ = 2.3 nM and 4.0 nM, respectively). Reformatting the Fabs into IgGs (Immunoglobulin Gs) increased their apparent affinities (notably, for H1 and H2, *K*_D_ = 0.5 nM and 0.3 nM, respectively), presumably due to the effects of avidity, with little change to their non-aggregation property. The six anti-hYKL-40 IgGs were analyzed using a trans-well migration assay in vitro, revealing that three clones (H1, H2, and H4) were notably effective in reducing cell migration from both A549 and H460 lung cancer cell lines. The three clones were further analyzed in an in vivo animal test that assessed their anti-cancer activities, demonstrating that the tumor area and the number of tumor nodules were significantly reduced in the lung tissues treated with H1 (IgG). Given its high affinity and desirable properties, we expect that the H1 anti-hYKL-40 mAb will be a suitable candidate for developing anti-cancer therapeutics.

## 1. Introduction

YKL-40, also known as chitinase 3-like 1 (Chi3L1), is a 40 kDa secreted glycoprotein belonging to the family of chitinase-like proteins (CLPs) [1]. It is a highly conserved chitin-binding protein and its crystallographic three-dimensional structures reveal the typical fold of the chitinase family protein but lacks chitinase activity due to mutation of an essential amino acid residue in its catalytic domain [2]. YKL-40 is overexpressed in cancers and tumor-associated macrophages, and its serum levels are elevated in patients with metastatic cancers and in various chronic inflammatory diseases, implicating pathological role of YKL-40 in cancer progression, metastasis, and inflammation [1,3,4,5,6].

While many things remain to be understood regarding the molecular nature of its action mechanism, a number of studies have verified YKL-40 as a promising therapeutic target for the treatment of various cancers and inflammatory diseases [1]. In various cancer cells, the YKL-40 signaling cascades are triggered by the membrane receptors such as syndecan-1, integrin αvβ5, VEGF receptor 2, and RAGE, leading to the increased expression levels of VEGF, MMP9, CCL2, and CXCL2 through FAK and ERK1/2-MAPK activity which result in the elevation of angiogenesis and tumor proliferation [7,8,9]. YKL-40-inhibiting small compounds such as caffeine, theophylline, chitin (β-(1-4)-poly-N-acetyl D-glucosamine), and siRNA complex are known to be effective in reducing tumor growth and metastasis in diverse kinds of cancers through down-regulation of signaling pathways, including PI3K/AKT, STAT3, and NF-kB, downstream of YKL-40 [10,11,12,13,14,15]. YKL-40 is also associated with various inflammatory diseases including atheroscleorosis, liver injury, and rheumatoid arthritis. The pro-inflammatory signaling pathways such as STAT3, caspase 3, and NF-κB, are induced by YKL-40–mediated RAGE activation. Intriguingly, YKL-40 is able to regulate apoptotic cell death depending on the association of TMEM219 with IL13Rα2, a known YKL-40 cellular receptor. That is, apoptotic cell death is reduced through the activation of the Erk1/2 and Akt signaling pathway when YKL-40 binds a YKL-40 receptor complex composed of TMEM219 and IL13Rα2, while YKL-40 binding to IL-13Rα2 in the absence of TMEM219 induces apoptotic cell death by stimulating the Wnt/β-catenin signaling [16,17,18]. YKL-40-inhibiting shRNA and miR-590-3p are known to reduce rheumatoid arthritis via downregulation of IL-18 production through PI3K/AKT pathway, while a proteasome inhibitor, Bortezomib, is found to suppress the expression of YKL-40, resulting in the reduction of pro-inflammatory and pro-fibrotic factors via down-regulation of NF-kB pathway [19,20]. Moreover, YKL-40 is associated with Alzheimer’s disease (AD) which is one of the inflammatory diseases. The expression of YKL-40 is induced by pro-inflammatory cytokines such as IL-1β and IL-6 through a STAT3 signaling pathway in astrocytes and the inflammation-induced YKL-40 activates the MAPK, β-catenin, and NF-κB signaling pathways via RAGE [16,21]. K284-6111, a YKL-40 inhibitor, reduces the expression of neuroinflammatory genes such as COX-2, iNOS, and GFAP in AD animal model through downregulation of NF-kB pathway, resulting in attenuation of memory dysfunction [22]. In addition to YKL-40-inhibiting small compounds, anti-YKL-40 antibodies were also found to attenuate angiogenesis via inhibition of tumor vascularization in brain and breast cancers [23,24,25].

As antibody-based therapeutics, full-length monoclonal antibodies (mAbs) have proven successful as drugs and remain unrivaled so far in spite of their drawback such as costly and time-consuming production in mammalian cell lines. In fact, most approved mAbs and those in regulatory review are canonical IgG antibodies [26,27]. Among numerous methods to identify human mAbs, phage display is a powerful tool that enables to display proteins and peptides on the surface of phage, which can be applied to study protein-protein interactions, define epitopes, identify enzyme inhibitors, screen antibody libraries, and to identify agonists and antagonists of cellular receptors [28,29,30]. In particular, phage display antibody libraries, generated in either naïve or synthetic antibody (mostly in Fab (Antigen-binding fragment) or scFv forms) formats, have proven to be highly successful for the selection of human mAbs against wide ranges of therapeutic targets ranging from cancers and inflammatory diseases to infectious diseases [31,32,33,34,35,36,37,38,39].

In this study, we panned synthetic human Fab phage display libraries against human YKL-40 (hYKL-40) and obtained monoclonal antibodies (mAbs) with high affinities and desirable biophysico-chemical properties for hYKL-40. We also described their activities interfering migration and proliferation of cancer cells, resulting in a selection of a candidate mAb, H1. We anticipate the H1 mAb can be further developed as a promising biologic in various pathological circumstances including cancers.

## 2. Results

### 2.1. Selection of Human Anti-hYKL-40 Fabs

**Figure 1:**
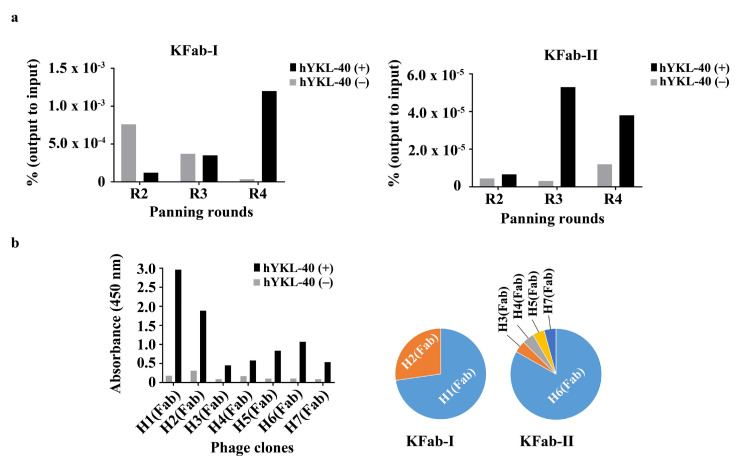
Panning of the phage-displayed synthetic Fab libraries on hYKL-40. (**a**) Monitoring of the phage titers over three rounds (R2–R4) of panning. Black and gray bars indicate the ratio of the phage output to the input titers, presented as a percentage (%), from panning on immobilized hYKL-4 (black, hYKL-40(+)) and non-immobilized hYKL-40 (gray, hYKL-40(−)) surfaces. The ratio of the output to the input (%) = (phage output titer ÷ phage input titer) × 100. (**b**) Phage ELISA performed on the immobilized hYKL-40 surfaces of seven Fab phage clones (left) and their selection frequency (%) over the panning (right). The selection frequency of a unique clone (%) = (Number of unique clones ÷ Total number of phage ELISA positives) × 100. Fab: Antigen-binding fragment; ELISA: enzyme-linked immunosorbent assay.

We have recently constructed some synthetic human antibody phage display libraries (size, ≈1 × 10^10^ for all): two synthetic human Fab phage display libraries (KFab-I and KFab-II, built on a human VH3/Vk1 and a VH1/Vk1 framework (FR), respectively) and a synthetic human scFv phage display library (KscFv-I, built on the same FR as the KFab-I library) (unpublished data). In order to isolate human antibodies that specifically recognize hYKL-40, the KFAb-I library was panned against a recombinant hYKL-40 immobilized on immunotubes, and 94 monoclonal phages from each of the third and fourth rounds of the panning were evaluated by ELISA (Figure 1a and Figure S1). Of the 94 individual clones from the third round, 26 had ELISA read-outs that were clearly higher than those of the background level (no immobilized hYKL-40 control) and were considered to be potential binders, while 86% (81 out of the 94) of the clones from the fourth round were determined to be positives based on their ELISA read-outs. The clones from the third round were sequenced, and 22 clones were determined to be complete and in-frame. The remaining clones contained mutations, such as frameshifts. By analyzing the CDR sequences of the 22 clones, 2 unique Fab clones (H1 (Fab) and H2 (Fab)) were identified. The H1 (Fab) was dominantly selected via panning (73% of sequences (16 out of the 22)), while the H2 (Fab) was present at a lower frequency (27% (6 out of the 22)) (Figure 1b). Since they already seemed quite enriched in the third round, the clones from the fourth round were not further analyzed (Figure S1).

In parallel, the other Fab phage display library, KFab-II, was also panned on a recombinant immobilized hYKL-40 surface, yielding 13 and 12 ELISA positives out of 94 individual clones from each of the third and fourth rounds of the panning, respectively, with relatively lower ELISA read-outs compared to those of the clones from the KFab-I library (Figure 1a and Figure S1). The clones were sequenced, and 23 clones were determined to be complete and in-frame. The remaining clones contained mutations, such as a stop codon (data not shown). By analyzing the CDR sequences of the 23 clones, 5 clones (H4 (Fab), H6 (Fab), and H7 (Fab) from the third round; H3 (Fab) and H5 (Fab) from the fourth round) were identified. H6 (Fab) was found to be the most dominant clone, with 83% of the sequences (19 out of 23), and the rest of the clones were selected by 1 out of the 23 clones (≈4%) (Figure 1b).

### 2.2. Production and Characterization of Human Anti-hYKL-40 Fabs

**Figure 2:**
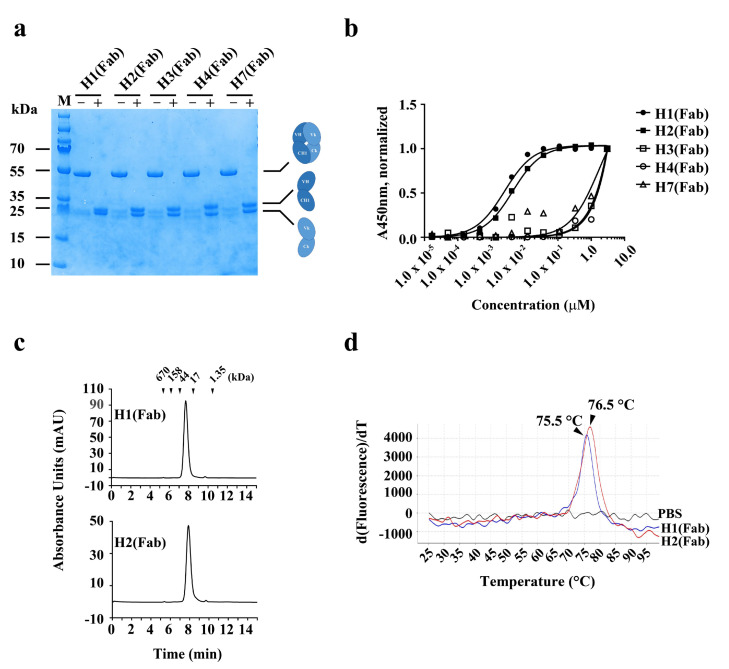
Production and characterization of human anti-hYKL-40 Fabs. (**a**) SDS-PAGE analysis of two human anti-hYKL-40 Fabs, H1 (Fab) and H2 (Fab), purified from periplasmic extracts of *E. coli* transformed with the indicated expression vectors. + and − indicate with and without the reducing reagent (β-mercaptoethanol), respectively. (**b**) Soluble ELISA of serially diluted H1 (Fab) and H2 (Fab) on immobilized hYKL-40 surfaces to measure their apparent affinities (*EC*_50_, nM). (**c**) Size-exclusion chromatography analysis of H1 (Fab) and H2 (Fab). The positions of the molecular mass markers, shown as kDa, on the retention time *x*-axis are shown above the peaks. (**d**) Protein thermal shift assay of H1 (Fab) and H2 (Fab) to determine their thermal stability (*T*_m_, °C). Fab: antigen-binding fragment; SDS-PAGE: sodium dodecyl sulfate-polyacrylamide gel electrophoresis; M: molecular mass marker.

To produce and characterize the binders as Fab proteins, the selected sequences were next cloned into an in-house bacterial expression vector (pKFAB). The Fabs were expressed in bacteria and subsequently purified. The resulting Fabs were highly pure, with protein yields of 1.8 mg/L, 0.2 mg/L, 0.2 mg/L, 0.5 mg/L, and 0.6 mg/L for H1 (Fab), H2 (Fab), H3 (Fab), H4 (Fab), and H7 (Fab), respectively (Figure 2a). H5 (Fab) and H6 (Fab) yielded little soluble protein.

The apparent affinities of the 5 Fabs for hYKL-40 were assessed using ELISA (*EC*_50_, nM) (Figure 2b). While the 3 Fabs (H3 (Fab), H4 (Fab), and H7 (Fab)) had very low apparent affinities for hYKL-40 (not fittable), the remaining Fabs, H1 (Fab) and H2 (Fab), showed notably higher apparent affinities (2.3 nM and 4.0 nM, respectively) (Figure 2b). The two Fab proteins H1 (Fab) and H2 (Fab) were observed to be monomeric, with no visible high-molecular-weight (HWM) aggregates found through size-exclusion chromatography (Figure 2c). In order to assess their thermal stability, the melting temperatures (*T*_m_, °C) of the 2 Fab proteins were measured using a protein thermal shift (PTS) assay. The results showed that H1 (Fab) and H2 (Fab) were stable, with a *T*_m_ of 75.5 °C and 76.5 °C, respectively (Figure 2d).

### 2.3. Production and Characterization of Human Anti-hYKL-40 IgGs

**Figure 3:**
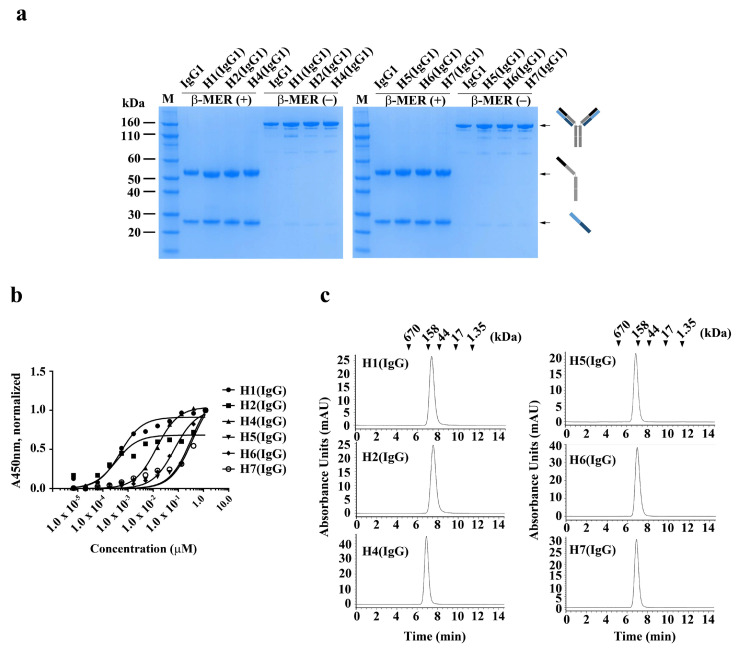
Production and characterization of anti-hYKL-40 IgGs. (**a**) SDS-PAGE analysis of three human anti-hYKL-40 IgGs, H1 (IgG), H2 (IgG), and H4 (IgG), purified from the culture media of Expi293F cells, which were transiently transfected with heavy- and light-chain expression vectors. β-MER (+) and β-MER (−) indicate with and without the reducing reagent (β-mercaptoethanol), respectively. (**b**) Soluble ELISA of serially diluted H1 (IgG), H2 (IgG), and H4 (IgG) on immobilized hYKL-40 surfaces to measure their apparent affinities (*EC*_50_, nM). (**c**) Size-exclusion chromatography analysis of H1 (IgG), H2 (IgG), and H4 (IgG). The positions of the molecular mass markers, shown as kDa, on the retention time *x*-axis are shown above the peaks.

In order to produce and characterize the Fab binders in an IgG form, the individual VH and VL sequences from each of the Fab clones were cloned into heavy (IgG1 Fc) and light chain (Ck1) expression vectors, respectively. IgGs were expressed transiently in Expi293 cells and subsequently purified. As shown in Figure 3, the resulting IgGs were highly pure, with protein yields of 0.8 mg/L, 5.9 mg/L, 21.5 mg/L, 1.0 mg/L, 1.7 mg/L, and 17.4 mg/L for H1 (IgG), H2 (IgG), H4 (IgG), H5 (IgG), H6 (IgG), and H7 (IgG), respectively (Figure 3a). H3 (IgG) could not be purified due to its aggregating nature.

To determine whether the apparent affinities of the IgGs for hYKL-40 were changed by reformatting the Fabs into IgGs, the apparent affinities of the IgGs were determined using ELISA (*EC*_50_, nM). As shown in Figure 3, the two clones, H1 (IgG) and H2 (IgG), which had high apparent affinities to the Fab forms, showed 5 to 14 fold-increased apparent affinities for H1 (IgG) and H2 (IgG), respectively, compared to their Fab formats (Figure 3b and Table 1), which is possibly due to an avidity effect [40]. The avidity effects were even more remarkable in the remaining IgGs. As shown in Table 1, the apparent affinity of H4 (IgG) was greatly increased to ≈14 nM from having ‘not fittable’ as its Fab form (Figure 3b), indicating that a significant avidity occurred. Next, to determine whether the IgGs were free of aggregates, they were analyzed using size-exclusion chromatography, revealing that the IgGs did not form any HMW aggregates (Figure 3b). H1 (IgG) was further analyzed using BLI (Bio-layer Interferometry) and a PTS (protein thermal shift) assay, showing that its apparent *K*_D_ and thermal stability (*T*_m_) were 5.0 × 10^−11^ M and 73.7 °C, respectively (Figure S2).

### 2.4. Trans-Well Migration Assay

**Figure 4:**
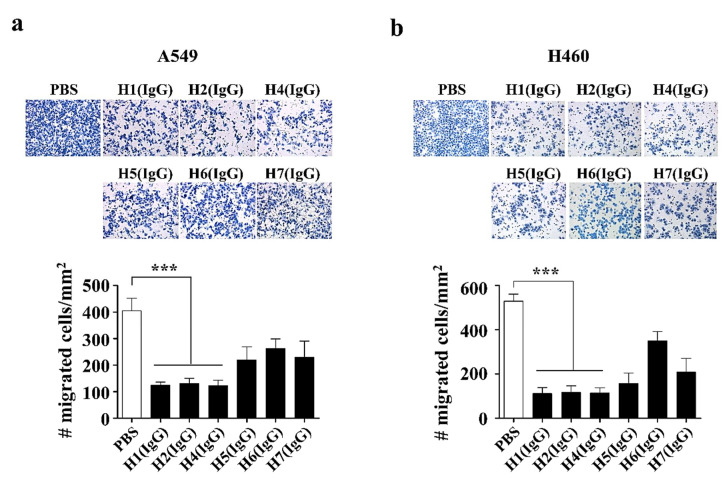
Trans-well migration assay of human anti-hYKL-40 IgGs. (**a**,**b**) Trans-well migration assay of human anti-hYKL-40 IgGs (H1 (IgG), H2 (IgG), H4 (IgG), H5 (IgG), H6 (IgG), and H7 (IgG)) on A549 (**a**) and H460 (**b**) cells to assess their inhibitory effects in cell migration. The number of migrated cells per square mm (mm^2^) was counted, plotted and compared to that of a negative control (PBS (phosphate-buffered saline)-treated). The data are presented as the mean ± SE (SEM). *** *p* < 0.001.

Based on our previous study, which revealed that the knock-down of YKL-40 suppressed cancer metastasis in lung cancer cell lines, A549 and H460) [41], we firstly wanted to know whether the anti-hYKL-40 IgGs could mimic the knock-down phenotype by antagonizing the activity of hYKL-40 in cancer cell migration. To address this, we adopted an in vitro trans-well migration assay to investigate their anti-metastatic activity in two different human lung cancer cell lines, A549 and H460 (Figure 4a,b, respectively). We found that all 6 anti-hYKL-40 IgGs significantly inhibited the migration in both the A549 and H460 cell lines. As shown in Figure 4a, 405 ± 21 cells/mm^2^ migrated to the lower side of the trans-well in the PBS-treated control group of A549 cells, while 124 ± 5, 132 ± 8, 124 ± 9, 220 ± 22, 263 ± 16, and 229 ± 27 cells/mm^2^ migrated to the H1 (IgG)-, H2 (IgG)-, H4 (IgG)-, H5 (IgG)-, H6 (IgG)-, and H7 (IgG)-treated groups, respectively. In Figure 4b, 529 ± 14 cells/mm^2^ migrated to the lower side of the trans-well in the PBS-treated control group of H460 cells. However, 112 ± 12, 117 ± 14, 114 ± 11, 158 ± 20, 349 ± 20, and 208 ± 28 cells/mm^2^ migrated to the H1 (IgG)-, H2 (IgG)-, H4 (IgG)-, H5 (IgG)-, H6 (IgG)-, and H7 (IgG)-treated groups, respectively. In order to further the in vivo study, we selected 3 anti-hYKL-40 IgGs (H1 (IgG), H2 (IgG), and H4 (IgG)), which showed a significantly higher degree of inhibition in the migration of the lung cancer cells. The 6 anti-hYKL-40 IgGs were further characterized on B16F10 mouse melanoma cells to assess their inhibitory effects in the migration of the melanoma cells prior to an in vivo assay on B16F10 cells. It was revealed that they all inhibited the migration in B16F10 cells as observed on the human lung cancer cells among which the 3 anti-hYKL-40 IgGs (H1, H2, and H4) were playing better compared to the others in antagonizing the cell migration (Figure S3).

### 2.5. In Vivo Anti-Cancer Effect of hYKL-40 IgGs

**Figure 5:**
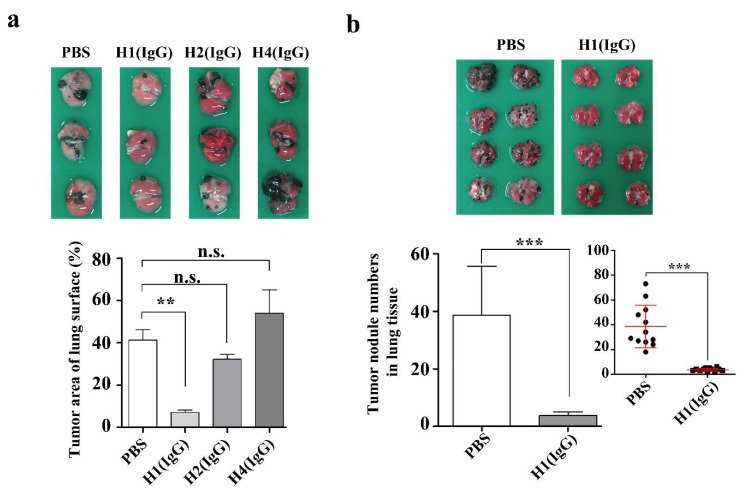
Anti-metastatic effect of anti-hYKL-40 IgGs. (**a**) A plot comparing the inhibitory effects of H1 (IgG), H2 (IgG), and H4 (IgG) by the tumor area on lung tissue. (**b**) A plot revealing the inhibitory effect of H1 (IgG) by the number of tumor nodules on the lung tissue. The original plot, showing the mean value and the standard error of the mean (SEM) marked in red lines, is shown as the inlet. The closed circles indicate the individual lung tissues examined. ** *p* < 0.01, *** *p* < 0.001. n.s.: not significant.

To investigate the in vivo anti-cancer effect of the three anti-hYKL-40 IgGs (H1 (IgG), H2 (IgG), and H4 (IgG)), which showed significant inhibitory activities in the in vitro assay on the human lung cancer cells and a mouse melanoma cell (B16F10) (Figure 4 and Figure S3), we considered the differences between applying and not applying the treatments of the anti-hYKL-40 IgGs, in terms of the tumor area and the number of tumor nodules on the lung tissues, to mice injected with B16F10 mouse melanoma cells (Figure 5). To address this, we measured the area of the tumor and total lung tissues of each mouse using a calipus, as described in the materials and methods. As shown in the Figure 5a, the tumor occupied 41.3 ± 5.0% of the surface of the lung tissues in the PBS-treated control group (with no treatment of anti-hYKL-40 IgG), while the tumor area on the surface of the lung tissues was significantly decreased to 7 ± 1.2% with the treatment of H1 (IgG). Unlike H1 (IgG), however, the tumor areas of the lung surfaces treated with H2 (IgG) and H4 (IgG) were 32.3 ± 2.2% and 54 ± 11.0%, respectively, which are not significantly different from the percentages of the PBS-treated control group. Furthermore, the average number of tumor nodules (38.7 ± 4.9) on the lung surface of the PBS-treated mice was significantly decreased to 3.8 ± 0.4 in H1 (IgG)-treated mice (Figure 5b). To see if H1 (IgG) is localized on the lung tissue, we performed an ex vivo imaging and revealed that H1 (IgG) is indeed present on the lung tissue (Figure S4). Moreover, a western blot analysis revealed that H1 (IgG) is able to recognize both recombinant human and mouse YKL-40s, and native YKL-40s from human and murine cells including human and mouse lung cells as well (Figure S5).

## 3. Discussion

We report the selection of human mAbs specific to hYKL-40 using human synthetic Fab phage display libraries. In vitro display technologies, such as phage and yeast display, have been successful for therapeutic applications against a variety of target antigens [36,42,43]. In particular, phage display is a powerful tool that has been proved to be highly effective for the selection of human antibodies through diverse human phage display libraries, mostly in either a naïve format generated from human peripheral blood mononuclear cells (PBMCs) or a semi-synthetic format constructed on a selected VH and VL scaffold by randomizing their CDRs [31,32,33,34]. Given the increased therapeutic value of YKL-40, many studies employing antibodies targeting hYKL-40 have been performed and reported that the antibodies are effective to modulate the biological processes that YKL-40 is involved, such as growth, differentiation, and metastasis of cancer cells [23,24,25] but most of the studies were performed with antibodies from mouse hybridoma and thus this is believed to be the first report of human antibodies targeting hYKL-40 identified from a human synthetic antibody phage display library. Moreover, human antibodies had desirable biophysical properties in terms of affinity, thermal stability, and non-aggregation, which are essential for the development of therapeutics.

Over the antibody selection with the phage display libraries employed, the KFab-I library, an in house human synthetic Fab phage display library constructed on a V_H_3 and a V_k_1 framework by randomizing their CDRs, yielded two anti-hYKL-40 mAb clones (H1 and H2) with high affinity of which H1 was selected dominantly (~73%) and found to be effective to inhibit growth and migration of cancer cells. Although more binders were identified from KFab-II, the other human synthetic Fab phage display library built on a V_H_1 and a V_k_1 framework, those clones were far below the two binders from KFab-I in their affinity and efficacy. Since the two libraries were built with CDRs designed by the same randomization scheme, we reasoned that the framework could make the difference. Previous studies have shown that among human V_H_ families, human V_H_3 has the highest stability and yield of soluble protein and its germline usage is about 43% (out of 51 germline segments) which is far above other human V_H_ families [44]. Indeed, it was revealed that a considerable number of antibodies selected from various antibody libraries including the Griffiths library and the HuCAL library belong to the V_H_3 family (74% for the Griffiths library and 36% for the HuCAL) [45,46]. This indicates that the V_H_3 framework is highly favored due to its desirable properties. However, since our phage display selections were performed separately with each Fab library but not with a mixture of both V_H_3 and V_H_1 frameworks from KFab-I and KFab-II, it is hard to say that the V_H_3 was indeed preferred to the V_H_1 framework for human YKL-40 over the phage display selections. More intriguingly, the scFv library we used did not yield any binder for hYKL-40 from the phage display selection. Since the scFv library is built on the same frameworks (V_H_3 and V_k_1) as the KFab-I library, it seems not due to the framework itself, but rather due to sub-optimal orientation of CDRs in the scFv form toward a binding region (i.e., epitope) on the human YKL-40 protein.

We showed that the H2, one of the two Fabs selected from KFab-I, had an affinity close to that of the H1 and had desirable properties as well, but the H2 turned out to be non-functional in the in vivo set of assay performed. Since the serum terminal half-life (t_1/2_) of human IgG1 in mice has been known to be about 9.5 days [47], we ruled out the possibility that it could be due to a short half-life of the human mAb in mice serum. Indeed, our pharmacokinetic analysis of H1 (IgG) in mice revealed a t_1/2_ of ~11 days which is comparable to the previous report (Figure S6 and Table S1) and we believe that other anti-hYKL-40 mAbs including H2 (IgG) and H4 (IgG) would have similar serum half-lives in mice as observed on H1 (IgG), since they all were built on the same framework (V_H_3 and V_k_1) and showed similar non-aggregation properties. Next, we reasoned that the two anti-hYKL-40 mAbs might recognize different epitopes so the epitope recognized by the H1 might play a more critical role in the neutralization. Indeed, an ELISA to test whether the two mAbs could compete on hYKL-40 immobilized on a surface revealed that the binding of H1 (Fab) with hYKL-40 seemed not to interfere with the presence of the H2 (Fab), suggesting that the two anti-hYKL-40 mAbs’ epitopes might not be overlapped (data not shown). However, we believe that this needs to be further confirmed with other methods such as BLI (Octet). We are currently undergoing to analyze the molecular detail of the association through a tertiary structure of the hYKL-40-H1 (Fab) complex using an X-ray crystallography (PDB deposit number 7CJ2), which should lead us to gain better understanding of how the association of the H1 with the epitope can lead to the anti-cancer activity. In addition, the structural understanding will possibly allow to further engineer the H1 to improve its affinity, and also to design and select small molecules including synthetic compounds and peptides. Moreover, recent studies showed that YKL-40 negatively regulates Th1 cells and cytotoxic T lymphocyte (CTL) activity and so its reduction by siRNA could increase the anti-cancer T cell population in lung metastasis [13], and somehow IL-13Rα2, a putative receptor of YKL-40, might play a role in their interplay with T cells [17,48]. Thus, it would be interesting to know if the H1 acts by antagonizing the interaction of YKL-40 with IL-13Rα2, and more interestingly how the H1 will interplay with YKL-40 in the T cell immunity in lung metastasis, which may shed a light on its possible interaction with PD-1, a well-known immune-checkpoint [13,49].

In conclusion, we have selected high-affinity human anti-hYKL-40 mAbs from human synthetic Fab phage display libraries. We characterized the resulting Fabs and IgGs to observe their desirable biophysical properties such as high affinity, non-aggregation, and thermal stability. We tested those in an in vitro and an in vivo assay to assess their anti-cancer activities and identified a clone, H1, which demonstrated its exceptional ability to inhibit growth and migration of cancer cells in vivo. Further refinement of the H1 mAb should warrant the development of a promising anti-cancer biologics.

## 4. Materials and Methods

### 4.1. Library Panning

Two human synthetic Fab phage display libraries produced in-house (KFab-I and KFab-II, built on a human VH3/Vk1 and a human VH1/Vk1 germline-based scaffold, with randomized complementarity-determining regions (CDRs), respectively) and a human synthetic scFv phage display library (KscFv-I, built on a human VH3/Vk1 germline-based scaffold, with randomized complementarity-determining regions (CDRs)) were used for the selection of specific binders against a recombinant human YKL-40 protein (hYKL-40) (Sino Biological, Beijing, China). hYKL-40 was immobilized in immunotubes (Nunc, Rochester, NY, USA) at a concentration of 10 μg/mL in PBS (phosphate-buffered saline, pH 7.4) at 4 °C for 18 h. After rinsing them twice with tap water, the immunotubes were blocked with 5% skim milk in PBS for 1 h at room temperature (RT). At the same time, the phage library was incubated in 2% skim milk in PBS for 1 h at room temperature (RT). Blocked phages were transferred to the immunotubes coated with hYKL-40 and incubated for 1 h at 37 °C, before being washed three times with PBS-T (PBS containing 0.05% Tween 20). Bound phages were eluted from immunotubes with 100 mM triethylamine for 10 min at RT, followed by neutralization with 1 M Tris-HCl (pH 7.4). Neutralized eluted phages were transferred to mid-log-phage *Escherichia coli* (*E. coli*) TG1 cells and incubated for 1 h at 37 °C with gentle rotation (120 rpm). The infected TG1 cells were spread on 2× YT agar plates supplemented with 2% glucose and 100 μg/mL ampicillin and incubated overnight at 37 °C. The colonies were collected by scraping with 6 mL of a 2× YT medium. A total of 50 mL of 2× YT supplemented with 2% glucose and 100 μg/mL ampicillin were inoculated with the scraped cells to yield an OD_600_ of 0.05 to 0.1 and incubated at 37 °C with shaking (220 rpm) until the OD_600_ reached 0.5. Then, the culture was infected with a VCSM13 helper phage (provided by Dr. Hong from Kangwon National University, Chuncheon-si, Gangwon-do, Korea) at a multiplicity of infection (M.O.I.) of 20:1. After incubation for 1 h at 37 °C with gentle rotation (120 rpm), kanamycin was added to a final concentration of 70 μg/mL, and the culture was grown overnight at 30 °C with shaking (220 rpm). Cells were then centrifuged at 24,793× *g* for 30 min, and the supernatant was passed through a 0.22 μm filter. Phage particles were precipitated using one-fifth of the volume of the precipitation buffer (20% PEG8000, 15% NaCl) for 30 min on ice. The precipitated phages were pelleted by centrifugation at 12,000 rpm for 30 min and resuspended in PBS. The phage suspension was used for the next round of panning. For stringent selections, the number of washing steps was gradually increased, and the amount of antigen for immobilization was decreased: first round: 10 μg/mL; second round: 5 μg/mL; third and fourth rounds: 1 μg/mL in PBS (1 mL).

### 4.2. Monoclonal Phage ELISA

A monoclonal phage ELISA was performed after three rounds of panning. Several 96-Well Half-Area Microplates (Corning, New York, NY, USA) were coated overnight at 4 °C, with 30 μL per well of 1 μg/mL hYKL-40, and each well was blocked with 5% skim milk in PBS for 1 h at RT. The amplified phages of individual clones from the third round of panning were added and incubated for 1 h at 37 °C. After washing four times with PBS-T, horseradish peroxidase (HRP)-conjugated anti-M13 antibody (1:5000, Sino Biological, Beijing, China) was incubated for 1 h at 37 °C. After washing it four times with PBS-T, a TMB substrate solution (Sigma-Aldrich, St. Louis, MO, USA) was added for 8 min, and the reaction was stopped with 1 N sulfuric acid (Merck, Darmstadt, Germany). The absorbance was measured at 450 nm using a SpectraMax 190 Microplate Reader (Molecular Devices, Sunnydale, CA, USA).

### 4.3. Production of Fab Proteins

An in-house bacterial expression vector (pKFAB) was used to construct the Fab expression vectors. The Fab fragments were amplified by a polymerase chain reaction (PCR). The PCR products were purified using a QIAquick PCR Purification Kit (QIAGEN, Hilden, Germany) and digested with *Sfi*I (New England Biolabs, Ipswich, MA, USA). The digestion products were separated on a 1% agarose gel, and the single band was purified with a QIAquick Gel Extraction Kit (QIAGEN, Hilden, Germany). The fragments were inserted into vector fragments digested using the same restriction enzymes with a T4 DNA ligase (New England Biolabs, Ipswich, MA, USA), and *E. coli* DH5α competent cells (Enzynomics, Daejeon, Korea) were transformed with the ligation mixtures. The individual colonies of the transformed cells were isolated, and the sequences of the isolated clones were verified. Top10F’ Competent Cells (Invitrogen, Carlsbad, CA, USA) were transformed with the Fab expression vectors, and the transformants were grown in 200 mL of TB (Terrific Broth) media supplemented with 100 μg/mL ampicillin at 37 °C with shaking (220 rpm) until the OD_600_ reached 0.5. The log-phase cultures were then induced with 0.5 mM isopropyl β-D-1-thiogalactopyranoside (IPTG) and incubated overnight at 30 °C with shaking (220 rpm). The cells were collected and resuspended in 16 mL of 1× TES (50 mM Tris-HCl, 1 mM EDTA, 20% Sucrose, pH 8.0). After incubation for 30 min on ice, 24 mL of 0.2× TES was added and incubated for 1 h on ice. The periplasmic fractions were collected after centrifugation at 12,000 rpm for 30 min and filtered through a 0.22 μm filter. The periplasmic extracts were loaded on a column packed with 0.5 mL of Strep-Tactin XT (IBA, Goettingen, Germany). The column was washed with 10 column volumes (CVs) of Buffer W (IBA, Goettingen, Germany) and eluted with 5 CVs of Buffer BXT (IBA, Goettingen, Germany). The eluted proteins were concentrated and buffer-exchanged with PBS using Amicon Ultra-15 Centrifuge Filter Units (Milipore, Carrigtwohill, Co., Cork, Ireland).

### 4.4. Determination of Apparent Affinity by ELISA

Several 96-Well Half-Area Microplates were coated overnight at 4 °C, with 30 μL per well of 2 μg/mL hYKL-40. After rinsing them twice with tap water, the wells were blocked with 5% skim milk in PBS for 1 h at RT. Serially diluted anti-hYKL-40 Fabs were added and incubated for 1 h at 37 °C. After washing the plates four times with PBS-T, the HRP-conjugated StrepMAB-Classic (1:10,000, IBA, Goettingen, Germany) was added to the plates and incubated for 1 h at 37 °C. After washing the plates four times with PBS-T, a TMB substrate solution was incubated for 8 min, and the reaction was stopped with 1 N sulfuric acid. The absorbance was measured at 450 nm using a SpectraMax 190 Microplate Reader. A plot was created using a non-linear curve fit algorithm with Graphpad Prism 7 (GraphPad Software, San Diego, CA, USA), and half-maximal effective concentration (*EC*_50_) values were determined accordingly.

### 4.5. Determination of Melting Temperature by a Protein Thermal Shift (PTS) Assay

To each well of a MicroAmp Fast Optical 96-Well Reaction Plate (Applied Biosystems, Foster City, CA, USA), 18 μL of anti-hYKL-40 Fab and 2 μL of Protein Thermal Shift Dye (10×, Applied Biosystems, Foster City, CA, USA) were added. As a negative control, PBS was mixed with the Protein Thermal Shift Dye. The plate was sealed with a MicroAmp Optical Adhesive Film (Applied Biosystems, Foster City, CA, USA) and centrifuged at 142× *g* for 1 min. The measurement was performed using a real-time PCR instrument. The instrument was set up according to the manufacturer’s instructions. All the experiments were performed at least in triplicate.

### 4.6. Size-Exclusion Chromatography (SEC) and Intact Mass Analysis

The separation of the antibodies using size-exclusion chromatography was conducted using a Waters Alliance 2695 (Waters, Milford, MA, USA) connected to a Biosuite high-resolution SEC column (7.5 mm × 300 mm, 10 µm particle size, Waters, Milford, MA, USA). The separation was performed using an isocratic elution with PBS, pH 7.4, at a flow rate of 1 mL/min. The effluent was detected using a UV/Vis detector 2489 at 280 nm.

### 4.7. Determination of Affinity by Bio-Layer Interferometry (BLI)

BLI (Bio-layer Interferometry) experiments were performed on an Octet QK384 (ForteBio, Menlo Park, CA, USA) instrument. The hYKL-40 protein was immobilized at 15 µg/mL in a 10 mM sodium acetate buffer (pH 5.0) and dispensed into a 96-well tilted-bottom microplate (200 µL per well) (Greiner bio-one, Monroe, NC, USA). Eight vertical wells were used at the same concentration. A second 96-well microplate contained the hYKL40-H1 antibody at 8 different concentrations (50 nM~0 nM, in 2-fold serial dilutions) and 1× PBS (supplemented with 0.09% (*v*/*v*) Tween 20) for baseline stabilization. Before the binding measurements, the AR2G (Amine reactive 2nd generation; ForteBio, Menlo Park, CA, USA) sensor tips were pre-hydrated in dH_2_O for 10 min, activated in a 1:1 mixture of 0.1 M N-Hydroxysuccinimide (NHS)/0.4 M 1-Ethyl-3-(3-dimethylaminopropyl)-carbodiimide (EDC) for 300 s and incubated in a binding buffer for 300 s (loading step). After a 180 s baseline dip in 1× PBS (supplemented with 0.09% (*v*/*v*) Tween 20), the binding kinetics were measured by dipping the hYKL-40-coated sensors into wells containing the H1 (IgG) antibody at various concentrations. The binding interactions were monitored over a 350 s association step, followed by a 500 s dissociation step, in which the sensors were dipped into new wells containing 1 × PBS (supplemented with 0.09% (*v*/*v*) Tween 20). Non-specific binding was assessed using sensor tips without the hYKL-40 protein. Data analysis was performed using Octet Data Analysis Software v6.4 (ForteBio, Menlo Park, CA, USA). The data were fitted to a 1:1 binding model to determine the association rate (*k*_a_) and dissociation rate (*k*_d_), and the equilibrium dissociation constant (*K*_D_, M) was calculated as follows: *K*_D_ = *k*_d_ ÷ *k*_a_.

### 4.8. Conversion to IgG and Production of IgG Proteins

The light- and heavy-chain vectors (pcDNA3.3 and pOptiVEC, respectively) used for the Herceptin expression were used as IgG1 backbone vectors. The VL, VH, and CL (Light-chain variable domain, heavy-chain variable domain, and light-chain constant domain, respectively) genes were individually amplified by a polymerase chain reaction (PCR), and then VL and CL were used in an overlap extension PCR. The PCR products (VL-CL and VH) were purified with a QIAquick PCR Purification Kit (QIAGEN, Hilden, Germany) and digested with the following restriction enzymes (New England Biolabs, Ipswich, MA, USA): VL-CL: *Sfi*I and *Eco*RI; VH: *Sfi*I and *Nhe*I. The digestion products were separated on a 1% agarose gel, and the single band was purified with a QIAquick Gel Extraction Kit (QIAGEN, Hilden, Germany). The fragments were inserted into vector fragments digested with the same restriction enzymes using a T4 DNA ligase (New England Biolabs, Ipswich, MA, USA), and *E. coli* DH5α competent cells (Enzynomics, Daejeon, Korea) were transformed with the ligation mixtures. Individual colonies of the transformed cells were isolated, and the sequences of isolated clones were verified.

Expi293F cells were cultured in an Expi293F Expression Medium (Thermo Fisher Scientific, Waltham, MA, USA) in a humidified 8% CO_2_ incubator at 37 °C with shaking at 125 rpm. On the day of transfection, the Expi293F cell density was approximately 2.9 × 10^6^ cells/mL. Expi293F transfections were performed using an ExpiFectamine 293 transfection reagent (Thermo Fisher Scientific, Waltham, MA, USA), according to the manufacturer’s protocol. IgG antibodies were purified using HiTrap MabSelect SuRe (GE Healthcare, Pittsburgh, PA, USA) columns. Briefly, equilibration was carried out using buffer A (PBS, pH 7.4). The sample was loaded onto the equilibrated column. Following sample loading, the column was washed with buffer A, until a stable baseline was established. Following the washing step, the protein was eluted with buffer B (0.1 M glycine, pH 2.7). Following the elution, the IgG was brought to a neutral pH using a 1 M Tris base, pH 9.0, and dialyzed into PBS gels (Thermo Fisher Scientific, Waltham, MA, USA). Purified and buffer-exchanged IgG samples were separated on 4–12% Bis-Tris gels (Thermo Fisher Scientific, Waltham, MA, USA) and stained with a Sun-Gel Staining Solution (LPS Solution, Deajeon, Korea).

### 4.9. Cell Culture

B16F10, A549, and H460 NSCLC cells were obtained from the American Type Culture Collection (Manassas, VA, USA). A549 and H460 cells were cultured in an RPMI 1640 medium supplemented with 10% heat-inactivated fetal bovine serum (FBS), 100 U/mL penicillin, and 100 μg/mL streptomycin. B16F10 skin melanoma cells were cultured in DMEM (supplemented with 10% heat-inactivated FBS, 100 U/mL penicillin, and 100 μg/mL streptomycin). Cell cultures were maintained in a humidified 5% CO_2_ incubator at 37 °C.

### 4.10. In Vitro Trans-Well Migration Assay

The migration of human lung cancer cells (A549 and H460) and mouse melanoma cells (B16F10) was quantitatively performed on permeable inserts (8 μm pore trans-well; Corning, New York, NY, USA). The cells treated with 1 μg/mL of anti-hYKL-40 IgGs were plated at 2.0 × 10^4^ cells per well (for A549 and H460) and 2.5 × 10^5^ cells (for B16F10) per well and incubated in a humidified 5% CO_2_ incubator at 37 °C for 17 h. After incubation, the cells were fixed with 3.7% formaldehyde for 2 min and then washed with PBS twice. Next, the cells were permeated with 100% methanol for 15 min at RT and stained with trypan blue for 20 min. Non-migrated cells on the inside of the wells were removed with a cotton swab, and the images, captured under a light microscope (Olympus, Tokyo, Japan) at 200× magnification, were analyzed, using NIH ImageJ software (imagej.nih.gov/ij/download/).

### 4.11. In Vivo Anti-Tumorigenic Assay

The study was conducted on random-bred, 6–7-week-old male C57BL/6 mice, with a body weight of 24–28 g. Animals were maintained under controlled conditions of temperature and light (Light:dark, 10 h:14 h.). They were provided standard mice feed (procured from Daehan Biolink, Eumsung, Korea) and water ad libitum. To induce metastasis, B16F10 mouse melanomas were injected intravenously into tail vein (3.75 × 10^4^ tumor cells/200 μL in phosphate-buffered saline (PBS) with a 27-gauge needle) of 8-week-old C57BL/6 mice [50,51]. After 4 days, 0.5 mpk (mg per kg body weight) of anti-hYKL-40 IgGs were inoculated through intravenous injection and these injections were performed every week for three weeks enough to maintain the concentration of IgG during this study period. Mice were sacrificed by 4 weeks after the injection of B16F10 cells to investigate the metastatic tumor nodule number and tumor area on the lung surface. The percentage of tumor area on the lung surface was measured with 2 pictures taken back and forth using NIH ImageJ software. The diameter of tumor nodules counted in this study was over 0.2 mm. All protocols involving mice in this study were reviewed and approved by the Chungbuk National University Institutional Animal Care and Use Committee (IACUC) on the date of 13 March 2017 and complied with the Korean National Institute of Health Guide for the Care and Use of Laboratory Animals (CBNUA-1073-17-01).

### 4.12. Ex Vivo Imaging of ICG-Labeled H1 (IgG)

H1 (IgG) was conjugated to ICG (Indocyanine green) using the ICG labeling kit (Dojindo, Kumamoto, Japan), according to the manufacturer’s instructions. ICG-labeled H1 (IgG) (1 mg/kg) was intravenously injected into C57BL/6 mice via the tail vein. Fifteen mins after administration, lungs were isolated and washed with PBS. Fluorescence intensity was analyzed by the VISQUE In vivo Optical Imager System (VIEWORKS, Gyeonggi, Korea). The NIR filter set (Excitation: 740 to 790 nm; Emission: 810 nm to 860 nm) was used for ICG fluorescence. A negative control was performed with lungs from PBS-treated mice.

### 4.13. Statistical Analysis

Statistical analysis was carried out using SPSS version 18.0 (IBM SPSS, New York, NY, USA). All error bars reported are the standard error of the mean (±SEM), unless otherwise indicated. Pairwise comparisons were conducted using a one-way Student’s *t*-test. Multiple comparisons were conducted using a one-way analysis of variance, followed by Tukey’s tests. Differences between groups are considered significant at *p*-values below 0.05 (* *p* < 0.05; ** *p* < 0.01; *** *p* < 0.001).

## 5. Conclusions

We have selected high-affinity human anti-hYKL-40 mAbs from human synthetic Fab phage display libraries. We characterized the resulting Fabs and IgGs to observe their desirable biophysical properties, such as their high affinity, non-aggregation, and thermal stability. We conducted in vitro and in vivo assays to assess their anti-cancer activities and identified a clone, H1, which demonstrated an exceptional ability to inhibit the growth and migration of cancer cells in vivo. A further refinement of the H1 mAb should allow for the development of a promising biological anti-cancer therapeutic.

## 6. Patents

We are in the process of obtaining a patent for the data on the human anti-hYKL-40 Fabs and IgGs in Korea (patent application number 10-2019-0112572; application date 11 September 2019).

## Figures and Tables

**Table 1:** Physicochemical properties of human anti-hYKL-40 monoclonal antibodies (mAbs).

Clones	Yield (mg/L Culture)	*T*_m_ (°C)	Monomericity (Mon/Agg.)	*EC*_50_ (nM)	*K*_D_ (M)
**H1 (Fab)**	1.8	76.5	Mon.	2.3	n.d.
**H2 (Fab)**	0.2	75.5	Mon.	4.0	n.d.
**H3 (Fab)**	0.2	n.d.	n.d	n.f.	n.d.
**H4 (Fab)**	0.5	n.d.	n.d	n.f.	n.d.
**H7 (Fab)**	0.6	n.d.	n.d	n.f.	n.d.
**H1 (IgG)**	0.8	73.7	Mon.	0.5	5.0 × 10^−11^
**H2 (IgG)**	5.9	n.d.	Mon.	0.3	n.d.
**H4 (IgG)**	21.5	n.d.	Mon.	13.6	n.d.
**H5 (IgG)**	1.0	n.d.	Mon.	327.4	n.d.
**H6 (IgG)**	1.7	n.d.	Mon.	69.4.	n.d.
**H7 (IgG)**	17.4	n.d.	Mon.	371.3	n.d.

Fab: Antigen-binding fragment; IgG: immunoglobulin G; n.d.: not determined; *T*_m_: melting temperature; *EC*_50_: half maximal effective concentration; *K*_D_: equilibrium dissociation constant; Mon.: monomer; Agg.: Aggregate; n.f.: not fittable.

## Supplementary Materials

Supplementary materials can be found at https://www.mdpi.com/1422-0067/21/17/6354/s1. Figure S1: Phage ELISA on an immobilized hYKL-40 surface with phages from each of the 3rd round and 4th round of the panning using KFab-I and KFab-II libraries; Figure S2. Determination of the affinity and melting temperature (*T*_m_) of H1 (IgG) using BLI (Octet); Figure S3. Trans-well migration assay of human anti-hYKL-40 IgGs on B16F10 melanoma cells; Figure S4. Ex vivo imaging of H1 (IgG) on the lung tissue; Figure S5. Western blot analysis on recombinant YKL-40 and cell lysates; Figure S6. The concentration-time profile of H1 (IgG) in C57BL/6 mice after intravenous administration (5 mg/kg); Table S1. Pharmacokinetic parameters of H1 (IgG).

## Abbreviations

mAb	Monoclonal antibody
CHI3L1	Chitinase-3-like 1
CLP	Chitinase-like protein
ELISA	Enzyme-linked immunosorbent assay
*EC*_50_	Half maximal effective concentration
IgG	Immunoglobulin G
Fab	Antigen-binding fragment
scFv	Single-chain variable fragment
SEC	Size-exclusion chromatography
PTS	Protein thermal shift
*T*_m_	Melting temperature
*K*_D_	Equilibrium dissociation constant
IPTG	Isopropyl β-D-1-thiogalactopyranoside
PBS	Phosphate-buffered saline
SDS-PAGE	Sodium dodecyl sulfate-polyacrylamide gel electrophoresis
CDR	Complementarity-determining region
FR	Framework
VH	Heavy-chain variable domain
VL	Light-chain variable domain
CL	Light-chain constant domain
BLI	Bio-layer Interferometry
