# Supplementary material for: Selection and Characterization of YKL-40-Targeting Monoclonal Antibodies from Human Synthetic Fab Phage Display Libraries
Source: Int J Mol Sci. 2020 Sep 1;21(17):6354. doi: 10.3390/ijms21176354 (PMC7504393; doi:10.3390/ijms21176354)
Supplement: Supplementary file 1 [file ijms-21-06354-s001.zip › ijms-848581-Supplementary Materials-to conversion/Table S1.pdf]

Table S1. Pharmacokinetic parameters of H1 (IgG). The pharmacokinetic parameters were measured after intravenous injections (5 mg/kg administration) with H1 (IgG) in C57BL/6 mouse.

Parameter	Value		
$t_{1/2}$ (day)	11.08	±	0.33
T_{max} (hr)	0.50	±	0.00
C_{max} (μg/mL)	92.21	±	22.22
C_{max}/dose (μg/mL/mg)	898.85	±	221.58
C_0 (μg/mL)	112.40	±	44.51
AUC_{last} (hr×mg/mL)	12.66	±	0.42
AUC_{last}/dose (hr×mg/mL/mg)	123.34	±	5.27
AUC_{inf} (hr×mg/mL)	13.01	±	0.45
AUC_{inf}/dose (hr×mg/mL/mg)	126.73	±	5.55
$AUC_{\%Extrap}$ (%)	2.67	±	0.21
V_d (mL/kg)	147.52	±	5.10
Cl (mL/hr/kg)	0.38	±	0.01

$t_{1/2}$, terminal half-life; T_{max}, time at maximal concentration; C_{max}, maximal concentration; C_{max}/dose, dose-normalized C_{max}; C_0, extrapolated zero time concentration; AUC_{last}, area under the curve from administration to the last measured concentration; AUC_{last}/dose, dose-normalized AUC_{last}; AUC_{inf}, area under the curve from administration to infinity; AUC_{inf}/dose, dose-normalized AUC_{inf}; $AUC_{\%Extrap}$, percentage of the extrapolated area under the curve at the total area under the curve; V_d, volume of distribution; Cl, clearance. Data presented as mean ± SD (total 6 mice, 3 mice/time point).
